# Role of prothrombin complex concentrate in perioperative coagulation therapy

**DOI:** 10.1186/s40560-014-0060-5

**Published:** 2014-10-29

**Authors:** Kenichi A Tanaka, Michael Mazzeffi, Miroslav Durila

**Affiliations:** Department of Anesthesiology, Cardiothoracic Anesthesia Division, University of Maryland, Suite S8D12, 22 South Greene Street, Baltimore, MD 21201 USA; Department of Anesthesiology and Critical Care Medicine, Second Faculty of Medicine, Charles University, Prague, Czech Republic

**Keywords:** Coagulation, Fresh frozen plasma, Prothrombin complex concentrate, Recombinant activated factor VII, Factor eight bypassing agent, Thromboelastometry

## Abstract

Prothrombin complex concentrate (PCC) is a term to describe pharmacological products that contain lyophilized, human plasma-derived vitamin K-dependent factors (F), FII, FVII, FIX, FX, and various amounts of proteins C and S. PCCs can be rapidly reconstituted in a small volume (20 ml for about 500 international units (IU)) at bedside and administered regardless of the patient’s blood type. PCCs are categorized as 4-factor PCC if they contain therapeutic amounts of FVII, and 3-factor PCC when FVII content is low. In addition, activated PCC which contains activated FVII and FX with prothrombin is available for factor VIII bypassing therapy in hemophilia patients with inhibitors. Currently, 4-factor PCC is approved for the management of bleeding in patients taking warfarin, but there has been increasing use of various PCCs in the treatment of acquired perioperative coagulopathy unrelated to warfarin therapy and in the management of bleeding due to novel oral anticoagulants. There is also an ongoing controversy about plasma transfusion and its potential hazards including transfusion-related lung injury (TRALI). Early fixed ratio plasma transfusion has been implemented in many trauma centers in the USA, whereas fibrinogen concentrate and PCC are preferred over plasma transfusion in some European centers.

In this review, the rationales for including PCCs in the perioperative hemostatic management will be discussed in conjunction with plasma transfusion.

## Introduction

Clinical management of severe bleeding in surgery and after trauma remains a major challenge. Transfusions of allogeneic platelets and plasma are widely used to replace cellular and humoral components of coagulation. These allogeneic products appear to be lifesaving when they are utilized early in the massive transfusion protocol in which more than six to ten units of packed red blood cells (RBCs) are typically administered in 12–24 h [1–3]. On the other hand, large amounts of allogeneic plasma have been associated with both infectious and non-infectious transfusion complications, which negatively impact patient survival [3–5].

Plasma-derived and recombinant factor concentrates are the lyophilized form of specific factor(s) indicated mainly for hereditary coagulation factor deficiencies [6]. The use of these factor concentrates in perioperative patients without hereditary coagulation disorders has been increasingly reported in anecdotal case series, retrospective, and prospective clinical studies [7,8]. Off-label use of recombinant activated factor VII (rFVIIa) has been rampant in the management of severe perioperative bleeding until several key clinical trials failed to improve outcomes and suggested a potential increase in thrombotic complications [9]. More recently, the FDA has approved the use of prothrombin complex concentrate (PCC) with therapeutic amounts of vitamin K-dependent factors for the management of bleeding in patients treated with warfarin [10]. For the latter, prothrombin, FVII, FIX, and FX are the specific ‘targets’ of PCC for replacement. Conversely, if plasma is used to reverse warfarin, multiple unnecessary elements are infused into the patient including albumin, antithrombin, fibrinogen, immunoglobulins, etc. Clinical indications, efficacies, and potential complications of plasma and factor concentrates should be carefully considered before they are applied to correct bleeding diathesis. Further, it is important to understand the mechanism of action of hemostatic agents and their impact on coagulation tests so that inappropriate dosing can be avoided.

The goal of this review is to discuss current limitations of plasma transfusion, pharmacology of PCCs, and their potential roles in surgical and trauma settings.

## Review

### Limitations of plasma

In the USA, two types of plasma are generally used for transfusion: fresh frozen plasma (FFP; plasma frozen within 8 h) and frozen plasma (FP24; plasma frozen at 8–24 h after collection). Once thawed, FFP and FP24 can be kept in the refrigerator (1°C–6°C) for 5 days, which potentially reduces plasma wastage and increases the inventories of group AB (universal) or group A plasma for emergency transfusion [11]. Although this has become a standard practice at large tertiary care centers in the North America, thawed plasma products may not be an option in smaller hospitals [12] and in other countries (Japan, Germany, etc.) [13].

Coagulation factor levels except for FVIII appear to stable for 5–7 days after thawing as long as plasma bags are kept at 1°C–6°C [13–15]. FVIII is most labile, and its activity falls by approximately 40% in 5 days. Some thawed plasma units (particularly group O plasma) may contain FVIII levels below 50% at the time of administration [14]. However, this is unlikely to have any clinical consequence because FVIII levels are often normal or elevated in most surgical and trauma patients [16,17]. Anticoagulant levels including antithrombin (AT) and protein C are also preserved, but protein S is decreased to 50% in 5-day old thawed plasma [13].

Although coagulation factor levels remain relatively stable in thawed plasma compared to FFP and FP24, there is a significant variability (50%–150%) in factor levels among individual donors [18,19]. For example, consider a case where one unit of plasma (250 ml) is being transfused to an 80-kg patient who currently has prothrombin activity of 20% (20 IU/dl). Assuming a plasma volume of 50 ml/kg, total amount of prothrombin in this patient is equal to:

$$ 80\mathrm{kg}\times 50\mathrm{ml}/\mathrm{kg}\times 0.2\mathrm{IU}/\mathrm{ml}=800\mathrm{IU} $$

If the donor plasma contains 80% prothrombin activity (80 IU/dl), the transfused amount of prothrombin is: 250 ml × 0.8 IU/ml =200 IU.

Thus, after transfusion (assuming 100% recovery), total prothrombin activity is:

$$ \left(800 + 200\right)\mathrm{IU}/\left(4,000+250\right)\mathrm{ml}=23.5\mathrm{IU}/\mathrm{dl}\left(23.5\%\right) $$

If four units of the same plasma are transfused, total prothrombin activity becomes:

$$ \left(800+200\times 4\right)\mathrm{IU}/\left(4,000+250\times 4\right)\mathrm{ml}=32.0\mathrm{IU}/\mathrm{dl}\ \left(32\%\right) $$

Similarly, if four units of another donor plasma with 120% prothrombin activity were transfused, total prothrombin activity becomes:

$$ \left(800+300\times 4\right)\mathrm{IU}/\left(4,000+250\times 4\right)\mathrm{ml}=40.0\mathrm{IU}/\mathrm{dl}\left(40\%\right) $$

Thus, plasma prothrombin activity is expected to be higher after the latter set of plasma, but normal prothrombin level above 50% is not achieved in this patient at the end of plasma transfusion (12.5 ml/kg). Indeed, it was previously reported that 30 ml/kg of plasma were needed to achieve 30% increase in procoagulant factor levels in critically ill patients with bleeding [20]. Large volumes of plasma transfusion increase not only the risk of transfusion-associated circulatory overload (TACO) but also the risk of transfusion-related acute lung injury (TRALI), a potentially lethal complication of plasma transfusion [21]. The pathogenesis of TRALI presumably involves leukocyte agglutination in pulmonary capillaries, which is triggered by donor antibodies against the recipient’s human leukocyte antigen (HLA) and neutrophil-specific antigens (HNA). Multiparous females are often sensitized to HLA antigens, and the recent switch to the preferential use male donor plasma has significantly reduced the incidence of TRALI to 1:12,000 [22]. However, in the emergency setting, about 40% of AB plasma is still from female donors, and the risk of TRALI is presumably higher [23]. It is also common to use ABO-compatible plasma, which is not identical to the recipient’s ABO group in the case of massive transfusion. In the retrospective matched cohort study of 568 trauma patients, the use of ABO-compatible plasma was dose-dependently associated with increased risks of acute respiratory distress syndrome and sepsis compared to the use of ABO-identical plasma [5].

Taken together, plasma transfusion should be implemented in the early phase of resuscitation for massive hemorrhage when bleeding sites are unknown, or no surgical control of bleeding has been achieved. Large volumes of required plasma (15–30 ml/kg) are usually tolerated because of hypovolemia due to hemorrhage. The risks of TRALI and other complications are theoretically increased after the exposure to multiple plasma units and donor antibodies. Newer commercial plasma products such as solvent-detergent plasma appear to have a minimal TRALI risk (i.e., anti-HLA/anti-granulocyte antibodies are significantly diluted by pooling of plasma from approximately 1,500 donors) [24], but it has not been widely used in the North America.

### PCCs versus plasma in acute warfarin reversal

PCC is a term used for lyophilized, human plasma-derived vitamin K-dependent coagulation factor concentrate. There are a number of commercial PCC products, and their availability varies among countries. According to the contents of coagulation factors, they are categorized into three groups: 4-factor PCC, 3-factor PCC, and activated PCC (Table 1).

**Table 1:** **Factor contents of commercially available PCCs**

	**FII IU/ml**	**FVII IU/ml**	**FIX IU/ml**	**FX IU/ml**	**PC IU/ml**	**PS IU/ml**	**Hepari U/ml**
4-factor PCC							
Beriplex/Kcentra (CSL Behring, Germany)	20–48	10–25	20–31	22–60	15–45	13–26	0.4–2
Octaplex (Octapharma, Austria)	11–38	9–24	25	18–30	7–31	7–32	<15
Prothromplex Total (Baxter, Austria)	24–45	25	30	30	>20	N.Q.	<15
PPSB-HT (Nichiyaku, Japan)	20	20	20	20	15–45	N.Q.	5
Cofact (Sanquin, Netherlands)	14–35	7–20	25	14–35	11–39	1–8	None
3-factor PCC							
Bebulin (Baxter, USA)	30	3–5	25	35	N.Q.	N.Q.	3.75
Profilnine (Octapharma, Austria)	37	3	25	16	N.Q.	N.Q.	None
Activated PCC							
FEIBA (Baxter, USA)	+	+ (FVIIa)	+	+ (FXa)	N.Q.	N.Q.	None

Other PCC products are also available in different countries. PCCs containing heparin are contraindicated in patients with heparin-induced thrombocytopenia. The potency of each PCC vial is according to the FIX activity except for FEIBA, which is dosed according to FVIII bypassing activity unit. Data are shown based on the prescribing information for each product; actual factor contents may vary for each vial.

*PC*/*PS* protein C/protein S, *N.Q*. not quantified.

The FDA approval of 4-factor PCC for acute warfarin reversal was based on the multicenter prospective randomized study of Kcentra® (CSL Behring, Marburg, Germany) and FFP in warfarin-treated adult patients (international normalized ratio (INR) >2.0) undergoing urgent surgery or invasive procedure [10]. This study demonstrated important differences in clinical endpoints and hematological parameters between 4-factor PCC and FFP. First, clinical hemostasis over 24 h with 4-factor PCC was non-inferior to FFP (72.4% vs. 65.4%, respectively). However, the median volume of infusion was much less for PCC than FFP (99.4 vs. 813.5 ml, respectively). Secondly, the target INR of ≤1.3 was reached after 30 min of intervention in 62.2% of patients treated with 4-factor PCC versus only in 9.6% of FFP-treated patients. It is also important to point out that the median time required to complete the treatment was much less for PCC than FFP (17.0 vs. 148 min, respectively).

**Figure 1:**
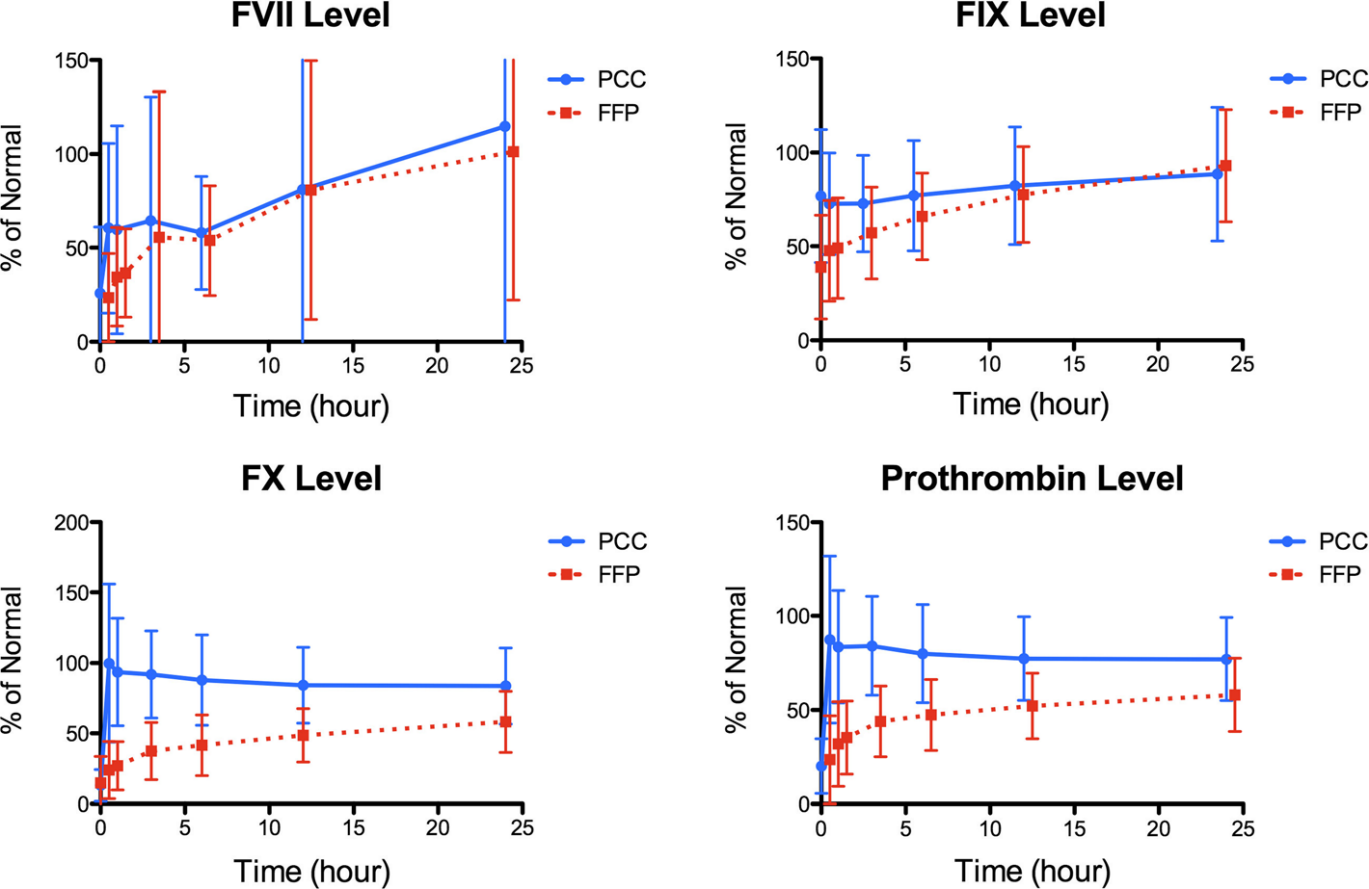
**Coagulation factor levels in warfarin-treated patients who received 4-factor PCC or plasma transfusion.** Mean plasma coagulation factor levels (% of normal activity ± standard deviation [SD]) for FVII, FIX, FX, and prothrombin (FII) are shown over 24 h after the injection of 4-factor PCC (4 F-PCC) or plasma transfusion. The elapsed time (h) from the treatment is indicated on the horizontal axis. Adapted from the reference [10] with permission.

Lastly, rapid recoveries of plasma vitamin K factor levels were clearly demonstrated within 30 min after infusing 4-factor PCC, while it took at least 3 h for plasma transfusion (and vitamin K) to bring procoagulant levels to ≥50% [10]. In particular, prothrombin (FII) and FX were recovered to 80%–100% of normal activity and maintained for 24 h with 4-factor PCC compared to plasma (Figure 1). Initial recoveries of FVII and FIX to 60%–80% were also better for 4-factor PCC than for plasma transfusion, but no difference between PCC and plasma was observed after 12 h.

3-factor PCC and activated PCC (factor eight bypassing agent; FEIBA) are specifically indicated for the prevention and treatment of hemophilia-related bleeding. However, 3-factor PCC is rarely used for this indication because human plasma-derived and recombinant FIX are available for hemophilia B. The use of 3-factor PCC for acute warfarin reversal has been reported prior to the approval of 4-factor PCC [24–27]. When INR values are very high (>5.0), 3-factor PCC is less effective than 4-factor PCC because FVII content is subtherapeutic in 3-factor PCC [24,28]. However, 3-factor PCC appears to be hemostatically effective due to high contents of FX and prothrombin [25–27]. Activated PCC (FEIBA) contains non-activated FII, FIX, and FX as well as trace amounts of FVIIa and FXa [29]. This agent is used in hemophilia patients with neutralizing antibodies to FVIII or FIX. The off-label use of FEIBA (500–1,000 IU) was shown to be more effective than 2–4 units of FFP in acute warfarin reversal [30].

The manufacturing process of PCCs from pooled human plasma includes pathogen reduction steps to prevent the transmission of lipid-enveloped viruses. These steps are solvent detergent exposure, pasteurization in the aqueous phase, or exposure of lyophilized product to vapor heat. There is a remaining concern for the transmissions of non-lipid enveloped, heat-resistant viruses including parvovirus B19 or variant Creutzfeldt-Jakob disease (vCJD; bovine spongiform encephalopathy (BSE)), and other emerging pathogens, but strict donor screening, nanofiltration of the source plasma, and inventory hold should sufficiently lower the risk of such transmissions [31].

### PCCs in acquired, non-warfarin coagulopathy

**Figure 2:**
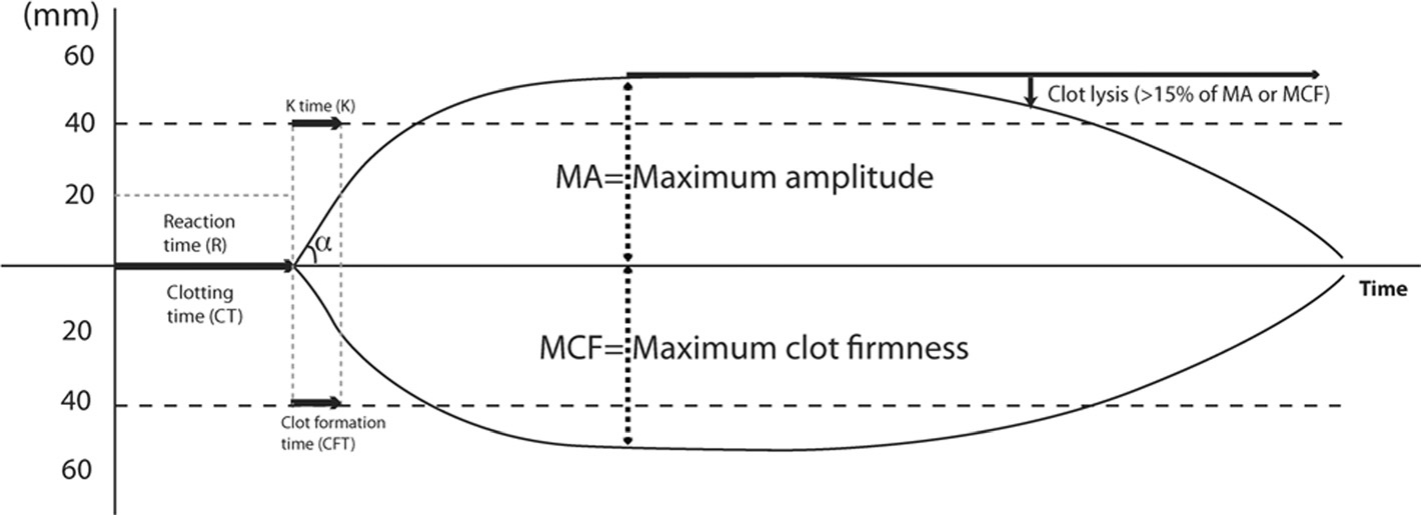
**Basic tracing patterns.** Changes in whole blood viscoelasticity are detected electromechanically in TEG® and optically in ROTEM®, and clot formation parameters are generated on TEG® (*top*) and ROTEM® (*bottom*). Plasmatic coagulation is reflected on R time and CT, initial clot development is shown on K time or CFT (also on α angle), and maximal viscoelasticity is defined by maximum amplitude (MA) or maximum clot firmness (MCF) for TEG® and ROTEM®, respectively. Systemic fibrinolysis is suspected when clot breakdown (>15% of MA or MCF) is observed within 1 h.

The FDA-approved indication for 4-factor PCC is limited to warfarin-related bleeding, but a number of publications have described the use of PCCs in acquired, non-warfarin coagulopathy in major trauma and surgery [32–35]. In contrast to warfarin anticoagulation in which only vitamin K-dependent factors are affected, perioperative coagulopathy often involves deficiencies of multiple coagulation factors and inhibitors. Multifactorial coagulation defects including thrombocytopenia, hypofibrinogenemia, and hyperfibrinolysis are important conditions that potentially decrease the efficacy of PCCs. These conditions can be quickly assessed by the use of whole blood viscoelastic tests. Thromboelastography (TEG®; Haemoscope-Haemonetics, Niles, IL) and thromboelastometry (ROTEM®, Durham, NC) are commercially available viscoelastic monitoring devices. Clot formation is assessed by the tensile (viscoelastic) force between the cup and the immersed pin resulting from the interaction between polymerizing fibrin and activated platelet glycoprotein (GP) IIb/IIIa receptors during endogenous thrombin generation and, if any, fibrin degradation by plasmin (Figure 2) [36].

In a retrospective study of 131 trauma patients (injury severity score, 38 ± 15), Schöchl et al. utilized a thromboelastometry-based protocol to initially restore fibrinogen to 150–200 mg/dl (target fibrinogen thromboelastometry [FIBTEM_MCF_ >10 mm]) and then corrected slow blood coagulation (i.e., enzymatic defect) by administering 4-factor PCC (target extrinsic thromboelastometry [EXTEM_CT_ ≤80 s]) [32]. Fibrinogen concentrate (median, 6 g or approximately 75.9 mg/kg) and 4-factor PCC (median, 1,800 IU or approximately 22.8 IU/kg) were respectively used in 96.2% and 73.7% of the subjects. On the other hand, platelet and plasma transfusions were given in 21.8% and 9.0% of the subjects, respectively. The overall mortality rate was 24.4%, which was considered to be lower than predicted mortalities (28%–34%) based on the injury scales.

In cardiac surgery with a moderate to high risk of postoperative bleeding, Weber et al. recently demonstrated that thromboelastometry-guided hemostatic intervention (*n* =50) was superior to the transfusion guided by conventional coagulation tests (control, *n* =50) [34]. In the intervention group, 4-factor PCC (25 IU/kg) was administered in the presence of slow blood coagulation (EXTEM_CT_ >80 s) and normal fibrin formation (FIBTEM_A10_ > 10 mm), while in the control group, plasma or PCC was given if INR >1.4 or after four units of RBCs were transfused when INR was unavailable. The overall use of PCC was similar between the two groups (44% vs. 52% in the control; *P* =0.433), but the incidence of plasma transfusion was significantly decreased in the intervention group (40% vs. 80% in the control; *P* <0.001). Similarly, there are two studies involving the deep hypothermic circulatory arrest, which demonstrated that plasma usage could be significantly decreased by thromboelastometry-guided hemostatic intervention compared to the conventional transfusion. Girdauskas et al. reported that incidences of plasma, fibrinogen, and 4-factor PCC usages (thromboelastometry vs. control) were 33% vs. 86% (*P* <0.001), 78% vs. 90% (*P* =0.7), and 15% vs. 90% (*P* <0.001) in aortic surgery (*n* =56) [37]. Similarly, Fassl et al. reported that plasma usage was 33% in the thromboelastometry group compared to 65% in the control (*P* =0.005) while there was no difference in the use of fibrinogen and 4-factor PCC between the two groups in aortic surgery [35]. Although sample sizes were relatively small (*n* ≤100) in the three cardiac surgery studies mentioned above, in-hospital adverse events including surgical reexploration, myocardial infarction, renal impairment, stroke, and death did not appear to be increased by the thromboelastometry-based intervention. In general, the overall incidence of PCC-related thromboembolic complications was reported to be 1.4% (95% CI, 0.8–2.1) in a recent meta-analysis of 27 studies (1,032 patients) including both 3-factor and 4-factor PCCs for the reversal of vitamin K antagonists [38]. Taken together, the abovementioned trauma and cardiac surgery studies suggested that factor concentrates can be effectively combined with allogeneic components using thromboelastometry-based protocols, resulting in reduced plasma transfusion [39].

### Reversal of novel oral anticoagulant

The use of novel oral anticoagulants (NOACs) is rapidly growing as an alternative to warfarin in venous thromboprophylaxis or stroke prevention in non-valvular atrial fibrillation. The direct thrombin inhibitor (anti-IIa), dabigatran, and the direct Xa inhibitor (anti-Xa) including rivaroxaban, apixaban, and edoxaban are prescribed without the need for coagulation testing. However, the lack of direct antidotes makes it difficult to manage their bleeding complications and to urgently reverse their effect for invasive procedures. Although there are specific neutralizing agents for anti-IIa and anti-Xa agents in clinical development [40,41], physicians presently struggle with managing bleeding complications of NOACs, particularly of dabigatran [42–45]. The use of rFVIIa and PCCs are considered to mitigate NOAC-associated bleeding, and they have been tested in animal and preclinical studies [46,47]. However, there are few data to support their clinical applicability. A case of massive transfusion related to dabigatran was reported in a patient undergoing aortic valve replacement and coronary artery bypass surgery. An extremely high-dose rFVIIa (21.6 mg or 270 μg/kg) was used in parallel with large amounts of blood products (plasma 22 units, cryoprecipitate 50 units, and platelets 5 adult doses), and bleeding was reported to be decreased. Hemostasis was finally achieved after several hours of hemodialysis for dabigatran removal. Another dabigatran-associated bleeding was reported after a cardiac perforation during an attempted atrial fibrillation ablation. Hypotension due to cardiac tamponade required epinephrine infusion, 8 l of fluid administration, six units of RBCs, and two units of FFP. After more than 4.5 l of blood was drained via pericardiocentesis, activated PCC (FEIBA, 26 U/kg) was intravenously given, and “notable slowing of bleeding” was observed by the cardiologist. Epinephrine infusion was subsequently stopped. There was another episode of hypotension 30 h after the procedure, the second dose of activated PCC (16 U/kg) was administered for suspected bleeding, and no thrombotic complication was observed. *In vitro* studies in human plasma suggest that both rFVIIa (120 μg/kg equivalent) and activated PCC (40–160 U/kg equivalent) improve the initiation of thrombin generation which is delayed by dabigatran [48]. Dabigatran-induced delayed thrombin generation was not improved by 4-factor PCC in human plasma [48,49], but several *in vivo* animal bleeding models support the use of 4-factor and 3-factor PCCs [50,51]. There are conflicting data regarding the use of PCCs in rivaroxaban-induced bleeding [52–54]. Taken together, the use of rFVIIa, PCC, and activated PCC may at least partially improve hemostasis in the presence of NOACs, but there is a paucity of clinical data to suggest a specific agent and dose. It is recommended that an emergency protocol for NOAC-associated bleeding is established at each institution by combining supportive measures and pharmacological interventions [55].

### Selecting plasma versus PCC in complex bleeding conditions

**Figure 3:**
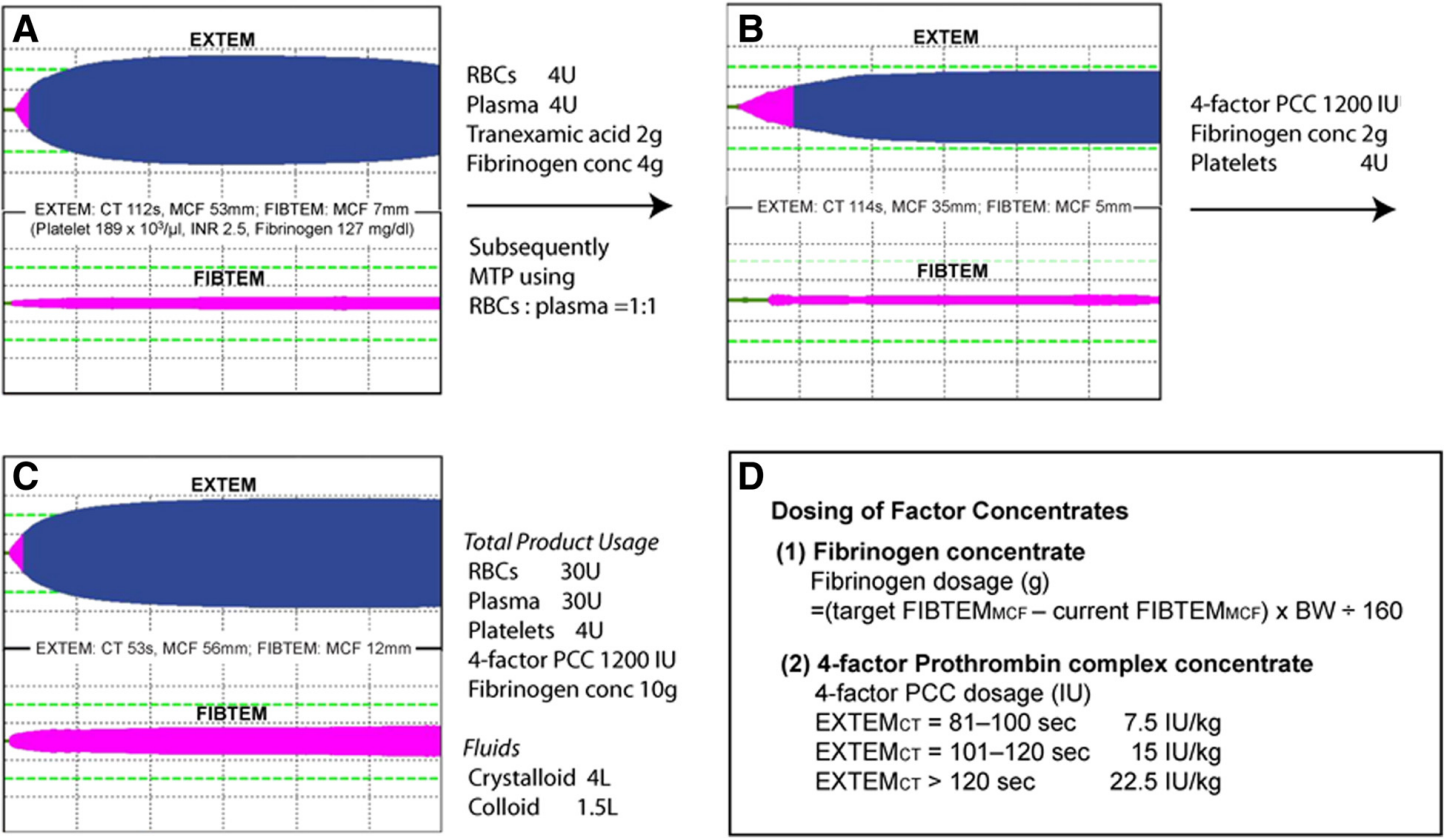
**Examples of rotational thromboelastometric tracings. (A)** Pre-treatment: traces obtained from a bleeding patient who incurred major trauma. Prolonged clotting time (CT) and late clot breakdown are notable on EXTEM. Fibrinogen level was on the borderline based on FIBTEM. Laboratory coagulation results were shown, but these results were not reported until the second ROTEM measurements were completed. Normal ranges for EXTEM and FIBTEM are as follows: EXTEM-CT 42–74 s, A_10_ 43–65 mm, and MCF 49–71 mm; FIBTEM-A_10_ 9–24 mm, MCF 9–25 mm. **(B)** On treatment: traces obtained after the initial treatment with 2 g of tranexamic acid and 4 g of fibrinogen along with ongoing 1:1 transfusion of RBCs and plasma. No improvement was seen in CT and MCF values of EXTEM. Fibrin polymerization was worsened on FIBTEM. **(C)** Normalized coagulation: traces obtained after the combined therapies using 1,200 IU of 4-factor PCC, 2 g of fibrinogen, and four units of platelets. Despite normal traces of EXTEM and FIBTEM, bleeding continued, and additional surgical hemostasis was established over the course of 8 h. The combination of allogeneic blood products and factor concentrates was crucial for managing massive volume depletion and ongoing bleeding. **(D)** Dosing of factor concentrates: the dose calculations for PCC and fibrinogen concentrate used in the protocol. BW = body weight. Adapted from the reference [56] with permission. MTP = massive transfusion protocol.

Clinical evidence and data supporting the use of factor concentrates in acquired coagulopathy have been increasing, but choosing this approach over plasma transfusion depends heavily on the clinical context. Although certain coagulation factors may be rapidly replenished by factor concentrates, they can be depleted again if there is no surgical control of bleeding. Durila and Malosek recently described the case of a 33-year-old woman who suffered from multiple traumatic injuries (ISS, 59) after a motor vehicle collision [56]. This patient required endotracheal intubation in the field and was brought to the hospital in hemorrhagic shock (pH 7.0, base excess −18.5 mmol/l) requiring norepinephrine to sustain blood pressure (70/40 mmHg). The obvious injury sites were an occipital open cranial wound and open fracture of the left thigh. In addition, free fluid was detected in the peritoneal cavity by ultrasound. The initial thromboelastometry traces showed delayed coagulation (EXTEM_CT_ 112 s; normal 35–80 s), late fibrinolysis on EXTEM, and borderline fibrinogen (FIBTEM_MCF_ 7 mm; normal 9–25 mm) (Figure 3A). The initial hemostatic intervention included 2 g of tranexamic acid, 4 g of fibrinogen (Haemocomplettan, CSL Behring, Germany), and four units of FFP and RBCs. The 1:1 ratio transfusion of FFP and RBCs was continued while intraabdominal and intracranial bleeding sources were sought and repaired by the surgical team. After an hour, the repeat thromboelastometry showed delayed coagulation (EXTEM_CT_ 114 s), thrombocytopenia (EXTEM_MCF_ 35 mm; normal 53–72 mm), and hypofibrinogenemia (FIBTEM_MCF_ 5 mm) (Figure 3B). The complex coagulopathy was managed with 1,200 units of 4-factor PCC (Prothromplex Total; Baxter, Austria), four units of random donor platelets, and an additional 4 g of fibrinogen. The combined hemostatic therapies normalized thromboelastometry parameters (Figure 3C,D), but the patient remained hemodynamically unstable, requiring continued norepinephrine infusion. This prompted the care team to look for another source of surgical bleeding, and splenic bleeding and intracranial bleeding sites were newly found by the contrast-enhanced computed tomography. After these were repaired, the patient became stable. Over the course of 8 h, 30 units of RBCs, 26 units of FFP, four units of platelets, 1,200 IU of PCC, and 10 g of fibrinogen were administered to this patient. In the end, the authors reported a favourable outcome of this case including no neurological deficit and no acute lung or kidney injury associated with blood transfusion [56]. Perioperative use of fibrinogen-rich components such as cryoprecipitate or fibrinogen concentrate has not been commonly used in Japan since plasma-derived (unheated) fibrinogen concentrate was recalled over hepatitis outbreak in the late 1980s [57]. However, Yamamoto et al. recently reported that the off-label use of (pathogen-inactivated) fibrinogen concentrate (*n* =25) in thoracic aortic replacement to maintain plasma fibrinogen level of 150 mg/dl was associated with a 58% reduction in the overall requirements for RBCs, FFP, and platelets when compared to the age-matched historical control (*n* =24) in which FFP and platelets were the only hemostatic measures [58]. Perioperative use of fibrinogen replacement can be considered as an adjunct to plasma transfusion, and the dosing can be optimized by fibrin-specific viscoelastic testing (Figure 3).

It may be argued that crystalloid and/or colloid should have been used in a complex perioperative bleeding case until surgical hemostasis is achieved instead of using allogeneic plasma. However, a large amount of crystalloid or colloid progressively worsens coagulation by dilutional effects [59] and possibly results in endothelial damage, tissue edema, and fluid overload [60]. Further, not all the coagulation factors and inhibitors can be replenished by currently available factor concentrates. Indeed, multiple microcapillary thrombi (in the lungs) and the signs of disseminated intravascular coagulation (DIC) were observed in four out of nine animals (44%) after a high dose (50 IU/kg) of 4-factor PCC (Cofact; Sanquin, Netherlands) in the presence of hepatic injury and hemodilution (70% of blood volume replacement) [61]. In their study, all nine animals that received a lower dose (35 IU/kg) of PCC had improved hemostasis (without DIC) and survival compared to the placebo-treated control animals. A fatal thrombosis in the left ventricle was reported in another porcine hepatic injury model with hemodilution (60%–70% of blood volume replacement) after coadministration of 200 mg/kg of fibrinogen (Haemocomplettan; CSL Behring, Germany) and 35 IU/kg of 4-factor PCC (Beriplex; CSL Behring, Germany) [62]. These preclinical data suggest that anticoagulant contents in 4-factor PCCs (mainly protein C and protein S) may be inadequate to reestablish the balance of procoagulant and anticoagulant elements in the setting of severe hemodilution (>70% of blood volume replacement). The 1:1 ratio transfusion of FFP and RBCs may not result in the establishment of hemostasis in the presence of massive bleeding. However, it is speculated that transfused plasma prevents excessive hemodilution of procoagulant and anticoagulant proteins [63,64], making subsequent procoagulant interventions (PCC and rFVIIa) more effective and less thrombogenic [65].

## Conclusions

The goal of hemostasis is to establish adequate fibrin polymerization at the site of vascular injury in a timely fashion. Hemostatic processes are obviously complex, involving vascular, humoral, and cellular components [66]. Perioperative coagulopathy often involves multifactorial defects in vascular (e.g., surgical bleeding), humoral, and cellular components. Transfusion algorithms based on thromboelastometry have become popular because this viscoelastic testing shows the extent of fibrin polymerization and the contributions of thrombin generation and platelet numbers to clot formation [36,67]. Timely diagnosis and therapeutic interventions are pivotal in restoring hemostatic function and hemodynamic stability in critically ill patients after trauma and major surgery [68]. The uses of PCCs and fibrinogen concentrate have advantages over allogeneic plasma in its rapid availability and restoration of target factor(s) and lower risks for volume overload and TRALI [10,69]. However, there should be a careful consideration in the dosing of PCCs because the risk of thrombosis may be enhanced in a subset of trauma and surgical patients [70] due to circulating tissue factor [17] and decreased plasma or endothelial anticoagulant activity [71]. In the early phase of hemostatic therapy after massive hemorrhage, plasma transfusion can be used to sustain procoagulant and anticoagulant factor levels. Once surgically correctable bleeding sites are found and nearly repaired, factor concentrate therapy can be implemented to optimize thrombin generation and fibrin polymerization under the guidance of thromboelastometry. In many countries, perioperative uses of PCCs and fibrinogen are considered as ‘off-label,’ but accumulating clinical data indicate that they are useful adjuncts in the management of preoperative coagulation.
